# The Chromatin Accessibility Landscape of Adult Rat

**DOI:** 10.3389/fgene.2021.651604

**Published:** 2021-05-24

**Authors:** Yue Yuan, Qiuting Deng, Xiaoyu Wei, Yang Liu, Qing Lan, Yu Jiang, Yeya Yu, Pengcheng Guo, Jiangshan Xu, Cong Yu, Lei Han, Mengnan Cheng, Peiying Wu, Xiao Zhang, Yiwei Lai, Giacomo Volpe, Miguel A. Esteban, Huanming Yang, Chuanyu Liu, Longqi Liu

**Affiliations:** ^1^BGI Education Center, University of Chinese Academy of Sciences, Shenzhen, China; ^2^BGI-Shenzhen, Shenzhen, China; ^3^First Hospital, Jilin University, Changchun, China; ^4^BGI College, Zhengzhou University, Zhengzhou, China; ^5^College of Veterinary Medicine, Jilin University, Changchun, China; ^6^Laboratory of Integrative Biology, Guangzhou Institutes of Biomedicine and Health, Chinese Academy of Sciences, Guangzhou, China; ^7^Bioland Laboratory (Guangzhou Regenerative Medicine and Health Guangdong Laboratory), Guangzhou, China; ^8^James D. Watson Institute of Genome Sciences, Hangzhou, China; ^9^Guangdong Provincial Academician Workstation of BGI Synthetic Genomics, BGI-Shenzhen, Shenzhen, China; ^10^Shenzhen Bay Laboratory, Shenzhen, China

**Keywords:** rat, body organs, brain regions, chromatin accessibility, transcription factors

## Introduction

The use of animal models has greatly facilitated biomedical discoveries to better understand human physiology and diseases (Baldini, 2010). For example, mice are widely used in biomedical research with advantages in fast breeding, easy feeding, and genetic engineering (Gurumurthy et al., 2020). Multi-omics datasets in mouse models [e.g., RNA-seq (Koh et al., 2016; Söllner et al., 2017; Han et al., 2018), ChIP-seq (Dahl et al., 2016; Liu et al., 2016; Zhang et al., 2016), and ATAC-seq (Assay for Transposase-Accessible Chromatin coupled with next-generation sequencing) (Wu et al., 2016; Cusanovich et al., 2018a; Liu et al., 2019)] are largely accumulating, at either tissue or single-cell levels, in both developmental (Dahl et al., 2016; Liu et al., 2016; Wu et al., 2016; Zhang et al., 2016) and pathological (Park et al., 2018) studies, further advancing our understanding of complex diseases. In contrast, while the Norway rat allows more precise modeling of complex human disorders [such as cardiovascular and psychiatric disease (Abbott, 2009; Jacob, 2010)], very few multi-omics studies have been performed on this model since the completion of the full genome sequencing of Brown Norway (BN) rat strain in 2004 (Gibbs and Pachter, 2004). In 2017, Söllner et al. (2017) were the first to conduct a comprehensive tissue-level transcriptome study on 13 tissues of rat and mouse tissues and found that, while the majority of genes are highly conserved, there are also few hundreds of genes that displayed opposite expression patterns in rats and mice. Hence, additional transcriptome and chromatin openness profilings would be of great importance to further unravel regulatory complexities of the rat genome.

In neuroscience, social behaviors such as grooming, sniffing, and chasing and their relationships with disease phenotypes have been assessed in great details in rat models (Meaney and Stewart, 1981; Schweinfurth, 2020). For example, a recent study has focused on both proteomic and transcriptomic profiling of the rat amygdala following a social play experiment and reported that the GABAergic, glutamatergic, and G-protein–coupled receptor signaling can be altered by social contexts (Alugubelly et al., 2019). By using rat as a model, future studies along this line would further illustrate mechanistic relationship between brain regions and social play behaviors.

Comprehensive omics datasets have paved the way for important discoveries in physiology, diseases, social behaviors, and many other areas. In this regard, previous studies have generated a collection of omics references (including genome, transcriptome, epigenome, and proteome) for model organisms such as human (Collins et al., 2003; Cao et al., 2020; Domcke et al., 2020), mouse (Cusanovich et al., 2018a; Han et al., 2018), worm (Gerstein et al., 2010; Li et al., 2014; Daugherty et al., 2017), and fly (Adams et al., 2000; Graveley et al., 2011; Cusanovich et al., 2018b). However, rat multi-omics data resources are still lacking. In this study, we comprehensively profiled the chromatin accessibility of 10 body organs (pancreas, adrenal gland, spleen, ovary, heart, ileum, lung, kidney, liver, thymus) and 12 different brain regions in female and male rats (somatosensory cortex, motor cortex, primary visual cortex, auditory cortex, prefrontal cortex, thalamus, cerebellum, striatum, hypothalamus, amygdala, hippocampus, olfactory bulb), by applying ATAC-seq (Buenrostro et al., 2013, 2015; Corces et al., 2017). This approach led to the identification of 397,691 chromatin accessible elements, with 34,219 body organ–specific peaks and 38,502 brain-specific peaks that were further characterized as tissue-specific regulatory elements. Interestingly, many of the enriched tissue-specific transcription factors were validated by previous studies (Liu et al., 2019). Here we provide a comprehensive rat tissue–specific chromatin accessibility landscape that would serve as an invaluable resource for future rat-related studies.

## Materials and Methods

### Sample Collection

Sprague–Dawley female and male rats, ~7–8 months old, were obtained from Jiangsu ALF Biotechnology Co., Ltd. (http://jsalfei.com) and used in the study. Experimental protocols related to the use of laboratory animals were approved by the Institutional Review Board on Ethics Committee of BGI (permit no. BGI-IRB A20020). Briefly, two female rats were sacrificed by carbon dioxide asphyxiation, and tissues were harvested and quickly frozen in liquid nitrogen and stored in liquid nitrogen, which include body organ tissues (e.g., pancreas, adrenal gland, spleen, ovary, heart, ileum, lung, kidney, liver, thymus) and brain regions (e.g., somatosensory cortex, motor cortex, primary visual cortex, auditory cortex, prefrontal cortex, thalamus, cerebellum, striatum, hypothalamus, amygdala, hippocampus, olfactory bulb). To assess gender-related variation, two male rats were also sacrificed, and the following organs were collected: pancreas, spleen, heart, lung, kidney, liver, thymus, cerebellum, epididymis, spermaduct, testis, hypothalamus, hippocampus, and olfactory bulb (Supplementary Table 1).

### Nuclei Isolation From Frozen Tissues

Nuclei were isolated from flash frozen rat tissues according to a standard nuclei extraction method (Corces et al., 2017). Briefly, shredded tissue sample was transferred to a prechilled 2 mL tissue Dounce homogenizer (Sigma, D8938-1SET) containing 2 mL of cold homogenization buffer [10 mM Tris-HCl, pH 8.0 (Sigma, T2694-1L), 25 mM KCl (Thermo, AM9640G), 5 mM MgCl_2_ (Ambion, AM9530G), 250 mM sucrose (BBI, SB0498), 0.1% NP40 (Roche, 11332473001), 0.1% Tween-20 (Sigma, P9416), 0.01% digitonin (Sigma, D141-100MG), 1 × protease inhibitor cocktail (Roche, 4693116001), and 0.1 mM DTT (Sigma, 646563) in nuclease-free water (Ambion, AM9932)]. At this stage, samples were incubated on ice for 5–10 min. Tissues were then homogenized with 10–15 strokes using pestle A (loose), followed by 20 strokes with pestle B (tight). The grinding and filtering parameters were adjusted, depending on the tissue type. Considering the possibility that density gradient centrifugation would specifically enrich certain types of nuclei, we skipped this step to avoid any sampling bias. After tissue grinding, nuclei suspensions were collected and centrifuged for 5 min at 500 × g in a 4°C prechilled swinging-bucket centrifuge. After removing the supernatant, nuclei pellets were washed twice with chilled wash buffer (10 mM Tris-HCl pH 8.0, 25 mM KCl, 5 mM MgCl_2_, 250 mM sucrose, 0.1% Tween-20, 1 × protease inhibitor cocktail, and 0.1 mM DTT in nuclease-free water). Then nuclei were counted by DAPI (Beyotime, C1006) staining.

### ATAC-Seq Library Construction

For the transposition step, we used 50,000 nuclei per each reaction. The transposition reaction mix contains 10 mM TAPS-NaOH (pH 8.5), 5 mM MgCl_2_, 10% DMF, 2.5 μL of in-house Tn5 transposase (0.8 U/μL) and phosphate-buffered saline (Gibco, 10010-031) (Buenrostro et al., 2015; Liu et al., 2019). Those reactions were incubated at 37°C for 30 min in a thermomixer shaking at 500 RPM. After transposition, the transposed DNA was purified with Qiagen MinElute PCR Purification Kit (Qiagen, 28006), and purified products were amplified with barcoded primers, NEBnext High-Fidelity PCR master mix (NEB, M0541S) as previously described. Libraries were quality controlled using Agilent High Sensitivity Assay (Agilent, 5067-4626). The library construction was considered successful upon confirmation of the correct average fragment size.

### Sequencing

All sequencing data were generated from MGISEQ-2000 platform (MGI). After size selection, libraries were quantified by Qubit dsDNA HS Assay Kit 3.0 (Invitrogen, Q32854). The polymerase chain reaction product from eight pooled libraries was heat-denatured and ligated into single-strand circular DNA. After DNB generation, libraries were then loaded on sequencing chip and sequenced with paired-end 50-bp reads (Huang et al., 2017).

### Preprocessing of the ATAC-Seq Datasets

After configuring the rat genome (rn6) and annotation files, the ATAC-seq raw data were processed by trimming, aligning, filtering, and quality controlling following an ATAC-seq pipeline from Kundaje lab (Koh et al., 2016). We used MACS2 (Zhang et al., 2008) (version 2.1.2) to identify peaks with options -B, -q 0.01, -nomodel, -f BAM. To evaluate the reproducibility between biological replicates of each tissue, we used the Irreproducible Discovery Rate method (Li et al., 2011) to identify overlapping peaks between replicates. Only the peaks present in both biological replicates were retained for subsequent analysis. We established the standard peak by merging overlapping peaks from all tissues, and we calculated the distance from nearest transcription start site (TSS) by using *distanceToNearest* function in GenomicRanges package (Lawrence et al., 2013). Then, we obtained the raw count matrix by using BedTools (Quinlan and Hall, 2010) (version 2.26.0) *intersect* function to count the reads mapped to each standard peak. We normalized the raw count matrix by reads per million mapped reads (RPM) and calculated Pearson correlation coefficients based on the log_10_ transformed RPM matrix.

### Identification of Tissue-Specific Chromatin Accessible Regions

Previously described strategy based on Shannon entropy (Schug et al., 2005; Barrera et al., 2008; Shen et al., 2012) was used to compute tissue-specific index. We defined the relative accessibility of each peak in a tissue type i as *Ri* = *Ei* / Σ*E*, where *Ei* is the RPM value of the peak in the tissue *i*, Σ*E* is the sum of RPM values in all tissues, and *N* is the total number of tissues. For each peak, the entropy score across tissues can be defined as *H* = −1 ^*^ sum(*Ri* ^*^ log_2_*Ri*) (1 < *i* < *N*), where the value of *H* ranges between 0 and log_2_(*N*). A highly tissue-specific peak owned an entropy score close to zero, while if a peak is conserved between different tissues, its entropy score close to log_2_(*N*) (Xie et al., 2013). Based on the distribution of entropy scores in our datasets, peaks with score <3 were identified as body organ–specific peaks, and peaks with score <2 were identified as brain region–specific peaks.

We searched TF motifs with the *findMotifsGenome.pl* script of the HOMER (Heinz et al., 2010) version 4.9.1 software and generated a motif enrichment matrix, where each row represents the *P*-value of a motif, and each column represents a tissue. We displayed the top 15 motifs of each tissue.

## Results

### ATAC-Seq Data Quality Control

**Figure 1:**
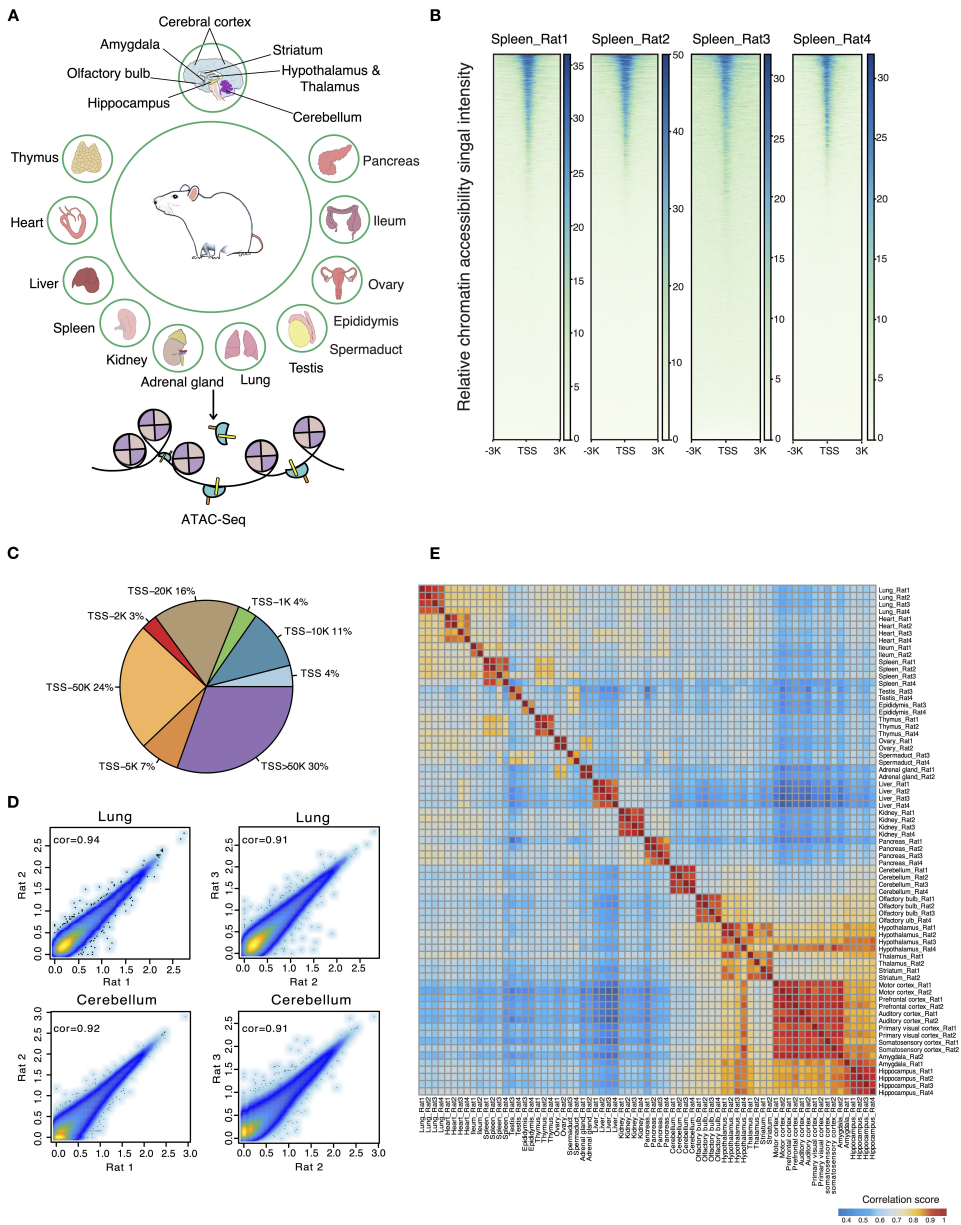
ATAC-seq data quality control and evaluation of reproducibility. **(A)** Thirty-six different tissues from adult female and male rats were collected for ATAC-seq profiling. **(B)** The ATAC-seq signal enrichment around TSSs for spleen samples (Rat1 and Rat2 represent female rats; Rat3 and Rat4 represent male rats). **(C)** Proportions of ATAC-seq peaks annotated to different genomic regions. **(D)** Scatter plots showing the Pearson correlations between biological replicates for two representative samples (lung and cerebellum of female and male rats). **(E)** Heatmap clustering across all 71 tissue ATAC-seq profiles.

A total of 72 frozen samples from 32 tissues were collected from two female and two male rats and used for bulk ATAC-seq profiling (Figure 1A). In total, we obtained an average of 77 million reads per sample and identified 34,219 body organ–specific and 38,502 brain-specific chromatin accessible elements from a total of 397,691 peaks (Supplementary Table 2). First, we systematically evaluated the quality of ATAC-seq datasets on several parameters including total raw read number, clean read number, mapping rate, and peak number (Supplementary Table 2). The enrichment score of reads at the TSS indicated our ATAC-seq datasets to be of high quality (Figure 1B). By calculating the proportions of ATAC-seq peaks annotated to different genomic regions, we observed the largest proportion of ATAC-seq peaks was annotated to distal peaks (Figure 1C), in agreement with the knowledge of distal regulatory elements being more abundant and important in ATAC-seq data (Buenrostro et al., 2013). Second, we evaluated the correlations between biological replicates obtained from either the same or opposite gender with Pearson correlation coefficients and observed high correlation. Specifically, the correlation between those of the same gender was subtly higher than that of the opposite gender (Figure 1D). As expected, heatmap clustering showed that tissue samples from the same organ were highly correlated, further demonstrating the reproducibility between biological replicates (Figure 1E). Furthermore, our analysis showed high correlations among five distinct cerebral cortex regions (motor, prefrontal, auditory, primary visual, and somatosensory cortex), whereas it showed lower correlations between brain regions of cerebellum, olfactory bulb, cerebral cortex, hippocampus, and other tissue types. In summary, our data survey reveals our rat ATAC-seq dataset to be of high quality and thus can be used to reliably detect chromatin accessible regions throughout the rat genome.

### Identification of Tissue-Specific Chromatin Accessible Peaks and Transcription Factors

**Figure 2:**
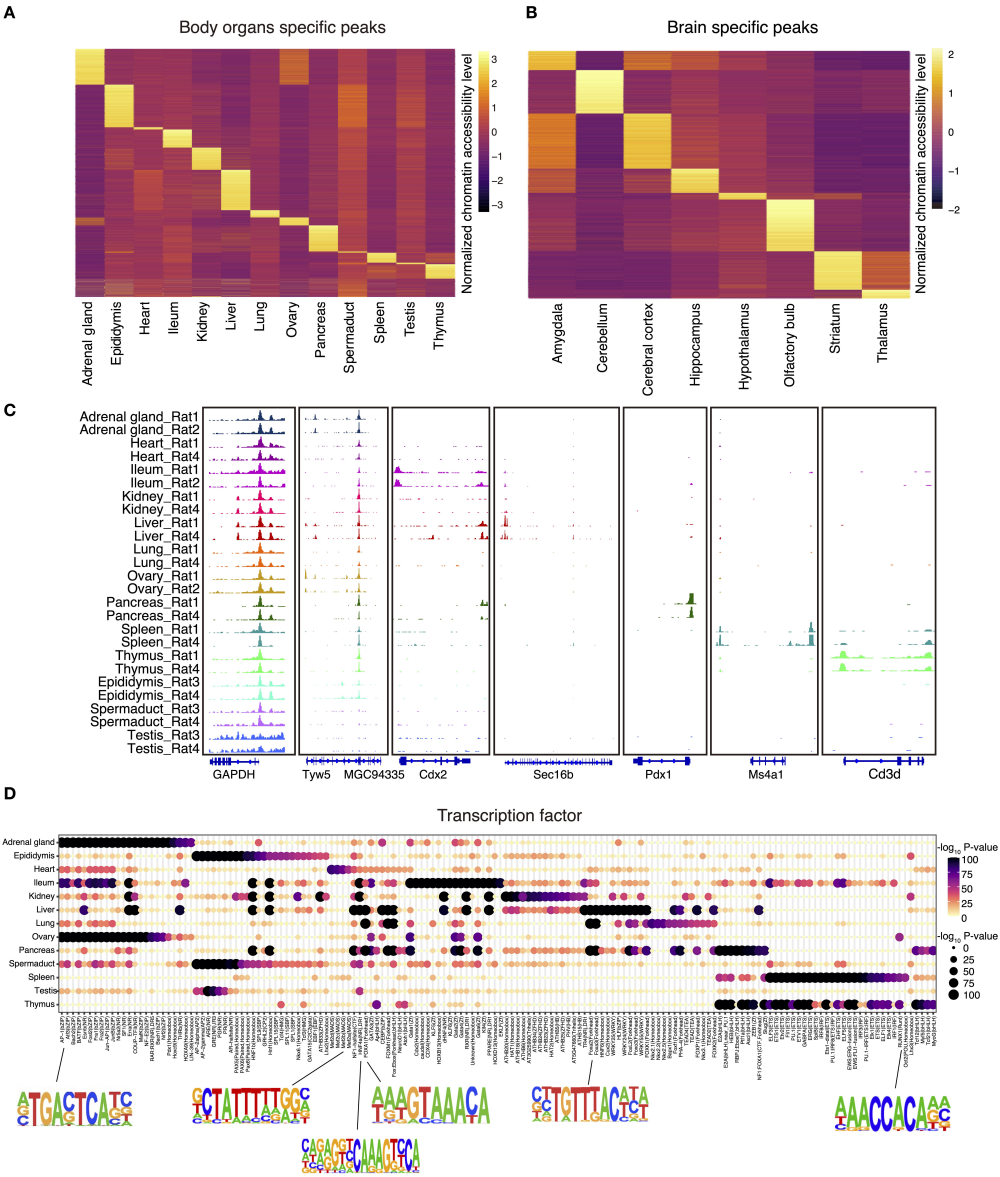
The landscape of tissue-specific chromatin accessibility and transcription factors. **(A)** Heatmap clustering showing the body organ–specific accessible elements. **(B)** Heatmap clustering showing the brain-specific accessible elements. **(C)** The integrative genomics viewer shows enrichment of ATAC-seq signal for the indicated housekeeping gene (*Gapdh*) and body organ–specific genes. **(D)** Enrichment of the indicated TF motifs in each tissue. The size and color of each point represent the motif enrichment *P*-value (–log_10_ *P*-value).

We next identified all genome-wide tissue-specific chromatin accessible regions as illustrated in Figures 2A,B. For tissue-specific genes, we provide integrative genomics viewer results showing higher ATAC-seq enrichment at annotated or putative promoters and enhancers (Figure 2C and Supplementary Figure 1A). For example, in spleen, ATAC-seq peaks are enriched around *Ms4a1*, which is a specific membrane protein gene expressed in B lymphocytes (Zuccolo et al., 2013) (Figure 2C). Similarly, analysis of the olfactory bulb revealed the enrichment of peaks near the *Cpa6* promoter, which is consistent with previous finding that *Cpa6* is highly expressed in the mitral and granular cell layers of olfactory bulb of adult mice as revealed by *in situ* hybridization (Fontenele-Neto et al., 2005) (Supplementary Figure 1A).

To verify the putative tissue-specific regulatory regions identified in our rat ATAC-seq data, we systematically compared those with existing studies from other species. By doing so, we found that tissue-specific transcription factors identified in rats are also highly consistent with those in other species. For example, previous studies have shown that HNF1b is an important transcriptional regulator of renal epithelial tissue (Ferrè and Igarashi, 2019), whereas HNF4a and HNF6a are key regulators during cellular differentiation in liver (Nagaki and Moriwaki, 2008). Our rat ATAC-seq atlas further confirmed that motifs of HNF1, HNF1b, HNF4a, HNF6, and HNF (hepatocyte nuclear factor) family transcription factors are enriched in liver, pancreas, ileum, and kidney in both rat and mice (Figure 2D). In addition, it has been reported that RUNX1 interplays with FOXL2 to maintain the identity of fetal ovary and secure the identity of ovarian-supporting cells (Nicol et al., 2019). Consistent with this, we found that RUNX1 motif is highly specific in rat ovary (Figure 2D). Furthermore, the MEF2 (myocyte enhancer factor 2, including MEF2a, MEF2b, MEF2c, MEF2d) family of transcription factors is a key regulator of cardiac muscle differentiation and development, as shown by the knockout of *Mef2a* gene, leading to dramatic changes of gene expression in the heart chamber (Medrano and Naya, 2017). MEF2a also plays a role in neuronal survival involved in memory and learning (Dietrich, 2013). Consistently, we also validated the enrichment of MEF2a in both heart and central neuron system in our rat atlas (Figure 2D and Supplementary Figure 1B).

In order to better understand the relationship between brain regions and social play behaviors, we also analyzed brain datasets to identify region-specific transcription factors. We first merged the datasets across different cerebral cortex for downstream region-specific peak analysis, as we did not observe significant differences in enriched transcription factors within cerebral cortex areas. A collection of brain region–specific peaks was identified among amygdala, cerebellum, cerebral cortex, hippocampus, and other regions (Figure 2B). In line with previous studies, we observed significant enrichments of NEUROG2, ATOH1, OLIG2, and NEUROD1 transcription factors in amygdala, cerebellum, cerebral cortex, and hippocampus (Supplementary Figure 1B). These transcription factors belong to the basic-helix–loop-helix (bHLH) family, and their function is crucial to determine the fate and differentiation of neural cells to ensure that different brain regions are supplied with the appropriate number of neuronal and glial cells (Dennis et al., 2019). Furthermore, transcription factor EGR1 has been shown to be related to the long-term fear memory and anxiety (Ko et al., 2005); our brain datasets unbiasedly identified EGR1 enrichment in the amygdala and hippocampus (the main brain areas for memory formation and storage), thus further validated previous findings.

## Conclusions

In summary, we have profiled the chromatin accessibility using ATAC-seq for 10 body tissues and 12 brain regions from adult rats and produced a large dataset with replicates. This comprehensive chromatin accessibility atlas contains 397,691 accessible elements. In addition, by comparing the open chromatin landscapes among rat tissues, a total of 34,219 body organ–specific peaks, 38,502 brain-specific peaks and a list of putative tissue-specific transcription factors were unbiasedly identified. We further showed that many known tissue-specific transcriptional characteristics can be recapitulated in this study, indicating that our data resource is of high quality and will be useful for future mechanistic discoveries in diseases and social behaviors.

## Data Availability Statement

All raw data have been submitted to the CNGB [Nucleotide Sequence Archive] [https://db.cngb.org/search/project/CNP0001470/] and also the NCBI [SRA] [https://www.ncbi.nlm.nih.gov/bioproject/PRJNA684678]. The ATAC-seq QC results have been submitted to Figshare under https://doi.org/10.6084/m9.figshare.13370498.v6.

## Ethics Statement

The animal study was reviewed and approved by Institutional Review Board on Ethics Committee of BGI.

## Author Contributions

YYua, QD, XW, CL, and LL conceived the idea. YJ, PG, and XZ collected samples. YYua, QD, and YYu generated the data. JX, PW, MC, and LH assisted with the experiments. XW analyzed the data with the assistance of YYua and YLi. YYua, wrote the manuscript with the input of XW. CL and LL supervised the study. LL, QL, GV, and CY revised the manuscript. YLa, ME, and HY provided helpful comments on this study. All authors reviewed and approved the final manuscript.

## Conflict of Interest

The authors declare that the research was conducted in the absence of any commercial or financial relationships that could be construed as a potential conflict of interest.

## Supplementary Material

Supplementary Figure 1 The landscape of brain-specific chromatin accessibility and transcription factors. **(A)** The integrative genomics viewer shows enrichment of ATAC-seq signal for the indicated housekeeping gene (*Gapdh*) and brain-specific genes. **(B)** Enrichment of the indicated TF motifs in each tissue. The size and color of each point represent the motif enrichment *P*-value (–log_10_ *P*-value).

Supplementary Table 1 Tissue and corresponding rat and sample IDs.

Supplementary Table 2 ATAC-seq metadata and mapping statistics.
