# Supplementary material for: The Chromatin Accessibility Landscape of Adult Rat
Source: Front Genet. 2021 May 24;12:651604. doi: 10.3389/fgene.2021.651604 (PMC8181391; doi:10.3389/fgene.2021.651604)
Supplement: Supplementary Figure 1 — The landscape of brain-specific chromatin accessibility and transcription factors. (A) The integrative genomics viewer shows enrichment of ATAC-seq signal for the indicated housekeeping gene (Gapdh) and brain-specific genes. (B) Enrichment of the indicated TF motifs in each tissue. The size and color of each point represent the motif enrichment P-value (–log10 P-value). [file Data_Sheet_1.ZIP › Supplementary/Supplementary Table 1. Tissue and corresponding rat and sample ID.docx]

| **Sample ID** | **Strain** | **Serial** | **Gender** | **Tissue** |
| --- | --- | --- | --- | --- |
| Adrenal gland_Rat1 | Sprague-Dawley | 1 | Female | Adrenal gland |
| Amygdala_Rat1 | Sprague-Dawley | 1 | Female | Amygdala |
| Auditory cortex_Rat1 | Sprague-Dawley | 1 | Female | Auditory cortex |
| Cerebellum_Rat1 | Sprague-Dawley | 1 | Female | Cerebellum |
| Somatosensory cortex_Rat1 | Sprague-Dawley | 1 | Female | Somatosensory cortex |
| Heart_Rat1 | Sprague-Dawley | 1 | Female | Heart |
| Hippocampus_Rat1 | Sprague-Dawley | 1 | Female | Hippocampus |
| Hypothalamus_Rat1 | Sprague-Dawley | 1 | Female | Hypothalamus |
| Ileum_Rat1 | Sprague-Dawley | 1 | Female | Ileum |
| Kidney_Rat1 | Sprague-Dawley | 1 | Female | Kidney |
| Liver_Rat1 | Sprague-Dawley | 1 | Female | Liver |
| Lung_Rat1 | Sprague-Dawley | 1 | Female | Lung |
| Motor cortex_Rat1 | Sprague-Dawley | 1 | Female | Motor cortex |
| Olfactory bulb_Rat1 | Sprague-Dawley | 1 | Female | Olfactory bulb |
| Ovary_Rat1 | Sprague-Dawley | 1 | Female | Ovary |
| Pancreas_Rat1 | Sprague-Dawley | 1 | Female | Pancreas |
| Prefrontal cortex_Rat1 | Sprague-Dawley | 1 | Female | Prefrontal cortex |
| Primary visual cortex_Rat1 | Sprague-Dawley | 1 | Female | Primary visual cortex |
| Spleen_Rat1 | Sprague-Dawley | 1 | Female | Spleen |
| Striatum_Rat1 | Sprague-Dawley | 1 | Female | Striatum |
| Thalamus_Rat1 | Sprague-Dawley | 1 | Female | Thalamus |
| Thymus_Rat1 | Sprague-Dawley | 1 | Female | Thymus |
| Adrenal gland_Rat2 | Sprague-Dawley | 2 | Female | Adrenal gland |
| Amygdala_Rat2 | Sprague-Dawley | 2 | Female | Amygdala |
| Auditory cortex_Rat2 | Sprague-Dawley | 2 | Female | Auditory cortex |
| Cerebellum_Rat2 | Sprague-Dawley | 2 | Female | Cerebellum |
| Somatosensory cortex_Rat2 | Sprague-Dawley | 2 | Female | Somatosensory cortex |
| Heart_Rat2 | Sprague-Dawley | 2 | Female | Heart |
| Hippocampus_Rat2 | Sprague-Dawley | 2 | Female | Hippocampus |
| Hypothalamus_Rat2 | Sprague-Dawley | 2 | Female | Hypothalamus |
| Ileum_Rat2 | Sprague-Dawley | 2 | Female | Ileum |
| Kidney_Rat2 | Sprague-Dawley | 2 | Female | Kidney |
| Liver_Rat2 | Sprague-Dawley | 2 | Female | Liver |
| Lung_Rat2 | Sprague-Dawley | 2 | Female | Lung |
| Motor cortex_Rat2 | Sprague-Dawley | 2 | Female | Motor cortex |
| Olfactory bulb_Rat2 | Sprague-Dawley | 2 | Female | Olfactory bulb |
| Ovary_Rat2 | Sprague-Dawley | 2 | Female | Ovary |
| Pancreas_Rat2 | Sprague-Dawley | 2 | Female | Pancreas |
| Prefrontal cortex_Rat2 | Sprague-Dawley | 2 | Female | Prefrontal cortex |
| Primary visual cortex_Rat2 | Sprague-Dawley | 2 | Female | Primary visual cortex |
| Spleen_Rat2 | Sprague-Dawley | 2 | Female | Spleen |
| Striatum_Rat2 | Sprague-Dawley | 2 | Female | Striatum |
| Thalamus_Rat2 | Sprague-Dawley | 2 | Female | Thalamus |
| Thymus_Rat2 | Sprague-Dawley | 2 | Female | Thymus |
| Cerebellum_Rat3 | Sprague-Dawley | 3 | Male | Cerebellum |
| Heart_Rat3 | Sprague-Dawley | 3 | Male | Heart |
| Hippocampus_Rat3 | Sprague-Dawley | 3 | Male | Hippocampus |
| Hypothalamus_Rat3 | Sprague-Dawley | 3 | Male | Hypothalamus |
| Kidney_Rat3 | Sprague-Dawley | 3 | Male | Kidney |
| Liver_Rat3 | Sprague-Dawley | 3 | Male | Liver |
| Lung_Rat3 | Sprague-Dawley | 3 | Male | Lung |
| Olfactory bulb_Rat3 | Sprague-Dawley | 3 | Male | Olfactory bulb |
| Pancreas_Rat3 | Sprague-Dawley | 3 | Male | Pancreas |
| Spleen_Rat3 | Sprague-Dawley | 3 | Male | Spleen |
| Thymus_Rat3 | Sprague-Dawley | 3 | Male | Thymus |
| Testis_Rat3 | Sprague-Dawley | 3 | Male | Testis |
| Epididymis_Rat3 | Sprague-Dawley | 3 | Male | Epididymis |
| Spermaduct_Rat3 | Sprague-Dawley | 3 | Male | Spermaduct |
| Cerebellum_Rat4 | Sprague-Dawley | 4 | Male | Cerebellum |
| Heart_Rat4 | Sprague-Dawley | 4 | Male | Heart |
| Hippocampus_Rat4 | Sprague-Dawley | 4 | Male | Hippocampus |
| Hypothalamus_Rat4 | Sprague-Dawley | 4 | Male | Hypothalamus |
| Kidney_Rat4 | Sprague-Dawley | 4 | Male | Kidney |
| Liver_Rat4 | Sprague-Dawley | 4 | Male | Liver |
| Lung_Rat4 | Sprague-Dawley | 4 | Male | Lung |
| Olfactory bulb_Rat4 | Sprague-Dawley | 4 | Male | Olfactory bulb |
| Pancreas_Rat4 | Sprague-Dawley | 4 | Male | Pancreas |
| Spleen_Rat4 | Sprague-Dawley | 4 | Male | Spleen |
| Thymus_Rat4 | Sprague-Dawley | 4 | Male | Thymus |
| Testis_Rat4 | Sprague-Dawley | 4 | Male | Testis |
| Epididymis_Rat4 | Sprague-Dawley | 4 | Male | Epididymis |
| Spermaduct_Rat4 | Sprague-Dawley | 4 | Male | Spermaduct |

**Supplementary Table 1.** Tissue and corresponding rat and sample IDs.
