# Supplementary material for: The Chromatin Accessibility Landscape of Adult Rat
Source: Front Genet. 2021 May 24;12:651604. doi: 10.3389/fgene.2021.651604 (PMC8181391; doi:10.3389/fgene.2021.651604)
Supplement: Supplementary Figure 1 — The landscape of brain-specific chromatin accessibility and transcription factors. (A) The integrative genomics viewer shows enrichment of ATAC-seq signal for the indicated housekeeping gene (Gapdh) and brain-specific genes. (B) Enrichment of the indicated TF motifs in each tissue. The size and color of each point represent the motif enrichment P-value (–log10 P-value). [file Data_Sheet_1.ZIP › Supplementary/Supplementary Table 2. ATAC-seq metadata and mapping statistics.docx]

| **Sample ID** | **Raw_Reads (Mb)** | **Clean_Reads (Mb)** | **Clean_Ratio (%)** | **Mapping_Ratio (%)** | **IDR Peaks** |
| --- | --- | --- | --- | --- | --- |
| Adrenal gland_Rat1 | 114 | 99.93 | 87.65 | 96.81 | 50640 |
| Amygdala_Rat1 | 73.36 | 62.33 | 84.98 | 97.85 | 42652 |
| Auditory cortex_Rat1 | 24.27 | 21.45 | 88.37 | 95.85 | 31917 |
| Cerebellum_Rat1 | 40.85 | 35.09 | 85.91 | 97.24 | 15454 |
| Somatosensory cortex_Rat1 | 26.47 | 22.39 | 84.56 | 96.66 | 29427 |
| Heart_Rat1 | 75.92 | 61.79 | 81.39 | 97.21 | 23854 |
| Hippocampus_Rat1 | 88.73 | 79.09 | 89.14 | 97.77 | 32223 |
| Hypothalamus_Rat1 | 75.47 | 64.75 | 85.8 | 97.52 | 35388 |
| Ileum_Rat1 | 88.03 | 72.58 | 82.44 | 97.48 | 17199 |
| Kidney_Rat1 | 41.1 | 33.85 | 82.36 | 95.64 | 32274 |
| Liver_Rat1 | 61.9 | 45.53 | 73.56 | 96.85 | 32922 |
| Lung_Rat1 | 99.47 | 88.25 | 88.72 | 97.80 | 23979 |
| Motor cortex_Rat1 | 46.38 | 36.73 | 79.18 | 96.61 | 32621 |
| Olfactory bulb_Rat1 | 63.44 | 52.71 | 83.08 | 96.84 | 26231 |
| Ovary_Rat1 | 80.88 | 73.26 | 90.58 | 97.10 | 51162 |
| Pancreas_Rat1 | 33.4 | 27.5 | 82.35 | 96.58 | 29381 |
| Prefrontal cortex_Rat1 | 42.37 | 37.39 | 88.26 | 97.17 | 53622 |
| Primary visual cortex_Rat1 | 41.14 | 33.9 | 82.39 | 96.08 | 23702 |
| Spleen_Rat1 | 85.48 | 68.73 | 80.4 | 97.12 | 22284 |
| Striatum_Rat1 | 37.69 | 29.95 | 79.46 | 96.71 | 26201 |
| Thalamus_Rat1 | 117.24 | 98.69 | 84.18 | 96.62 | 19452 |
| Thymus_Rat1 | 83.01 | 68.24 | 82.2 | 97.11 | 16881 |
| Adrenal gland_Rat2 | 81.12 | 68.17 | 84.03 | 97.15 | 50640 |
| Amygdala_Rat2 | 84.31 | 74.89 | 88.83 | 98.69 | 42652 |
| Auditory cortex_Rat2 | 72.13 | 62.68 | 86.9 | 91.41 | 31917 |
| Cerebellum_Rat2 | 23.08 | 19.2 | 83.19 | 96.75 | 15454 |
| Somatosensory cortex_Rat2 | 76.12 | 63.39 | 83.27 | 96.91 | 29427 |
| Heart_Rat2 | 90.49 | 62.94 | 69.56 | 97.62 | 23854 |
| Hippocampus_Rat2 | 107.37 | 86.8 | 80.85 | 98.67 | 32223 |
| Hypothalamus_Rat2 | 69.49 | 61.98 | 89.19 | 98.57 | 35388 |
| Ileum_Rat2 | 85.98 | 76.12 | 88.53 | 97.97 | 17199 |
| Kidney_Rat2 | 84.78 | 73.44 | 86.62 | 97.96 | 32274 |
| Liver_Rat2 | 102.9 | 85.91 | 83.49 | 97.95 | 32922 |
| Lung_Rat2 | 87.38 | 76.55 | 87.6 | 98.75 | 23979 |
| Motor cortex_Rat2 | 71.49 | 56.98 | 79.71 | 96.32 | 32621 |
| Olfactory bulb_Rat2 | 37.04 | 33.83 | 91.35 | 98.01 | 26231 |
| Ovary_Rat2 | 133.77 | 117.33 | 87.71 | 98.20 | 51162 |
| Pancreas_Rat2 | 82.14 | 69.06 | 84.08 | 97.15 | 29381 |
| Prefrontal cortex_Rat2 | 131.21 | 105.94 | 80.74 | 98.46 | 53622 |
| Primary visual cortex_Rat2 | 62.96 | 51.34 | 81.54 | 96.74 | 23702 |
| Spleen_Rat2 | 78.29 | 65.72 | 83.95 | 96.74 | 22284 |
| Striatum_Rat2 | 105.78 | 88.73 | 83.88 | 97.93 | 26201 |
| Thalamus_Rat2 | 29.34 | 24.66 | 84.07 | 96.24 | 19452 |
| Thymus_Rat2 | 32.72 | 30.22 | 92.36 | 98.02 | 16881 |
| Cerebellum_Rat3 | 109.15 | 94.27 | 86.37 | 98.21 | 32612 |
| Heart_Rat3 | 70.08 | 62.98 | 89.87 | 97.76 | 14927 |
| Hippocampus_Rat3 | 78.53 | 65.67 | 83.62 | 98.13 | 28297 |
| Hypothalamus_Rat3 | 80.89 | 72.28 | 89.36 | 98.56 | 19555 |
| Kidney_Rat3 | 87.06 | 77.2 | 88.67 | 98.22 | 32505 |
| Liver_Rat3 | 33.21 | 29.25 | 88.06 | 98.45 | 32046 |
| Lung_Rat3 | 58 | 53.33 | 91.95 | 96.48 | 18507 |
| Olfactory bulb_Rat3 | 69.75 | 60.92 | 87.34 | 98.45 | 18424 |
| Pancreas_Rat3 | 83.09 | 75.84 | 91.28 | 98.55 | 21855 |
| Spleen_Rat3 | 138.88 | 114.21 | 82.24 | 98.38 | 19516 |
| Thymus_Rat3 | 92.02 | 70.8 | 76.94 | 95.46 | 9206 |
| Testis_Rat3 | 91.37 | 88.07 | 96.38 | 98.67 | 5461 |
| Epididymis_Rat3 | 88.39 | 79.87 | 90.36 | 98.58 | 26463 |
| Spermaduct_Rat3 | 115.05 | 107.73 | 93.64 | 98.37 | 4714 |
| Cerebellum_Rat4 | 86.3 | 75.59 | 87.59 | 97.98 | 32612 |
| Heart_Rat4 | 110.35 | 95.09 | 86.18 | 98.46 | 14927 |
| Hippocampus_Rat4 | 85.01 | 75.51 | 88.82 | 98.44 | 28297 |
| Hypothalamus_Rat4 | 101.78 | 90.76 | 89.17 | 98.69 | 19555 |
| Kidney_Rat4 | 90.9 | 77.86 | 85.65 | 94.65 | 32505 |
| Liver_Rat4 | 45.4 | 37.21 | 81.95 | 98.59 | 32046 |
| Lung_Rat4 | 74.75 | 67.81 | 90.71 | 98.35 | 18507 |
| Olfactory bulb_Rat4 | 82.58 | 71.28 | 86.32 | 98.18 | 18424 |
| Pancreas_Rat4 | 108.42 | 101.55 | 93.66 | 98.72 | 21855 |
| Spleen_Rat4 | 47.45 | 40.05 | 84.39 | 97.81 | 19516 |
| Thymus_Rat4 | 72.56 | 64.06 | 88.29 | 97.82 | 9206 |
| Testis_Rat4 | 88.68 | 84.62 | 95.42 | 98.69 | 5461 |
| Epididymis_Rat4 | 85.86 | 79.28 | 92.33 | 98.56 | 26463 |
| Spermaduct_Rat4 | 136.94 | 129.24 | 94.38 | 98.19 | 4714 |

**Supplementary Table 2.** ATAC-seq metadata and mapping statistics.
