# Supplementary figures and images for: The Chromatin Accessibility Landscape of Adult Rat
Source: Front Genet. 2021 May 24;12:651604. doi: 10.3389/fgene.2021.651604 (PMC8181391; doi:10.3389/fgene.2021.651604)

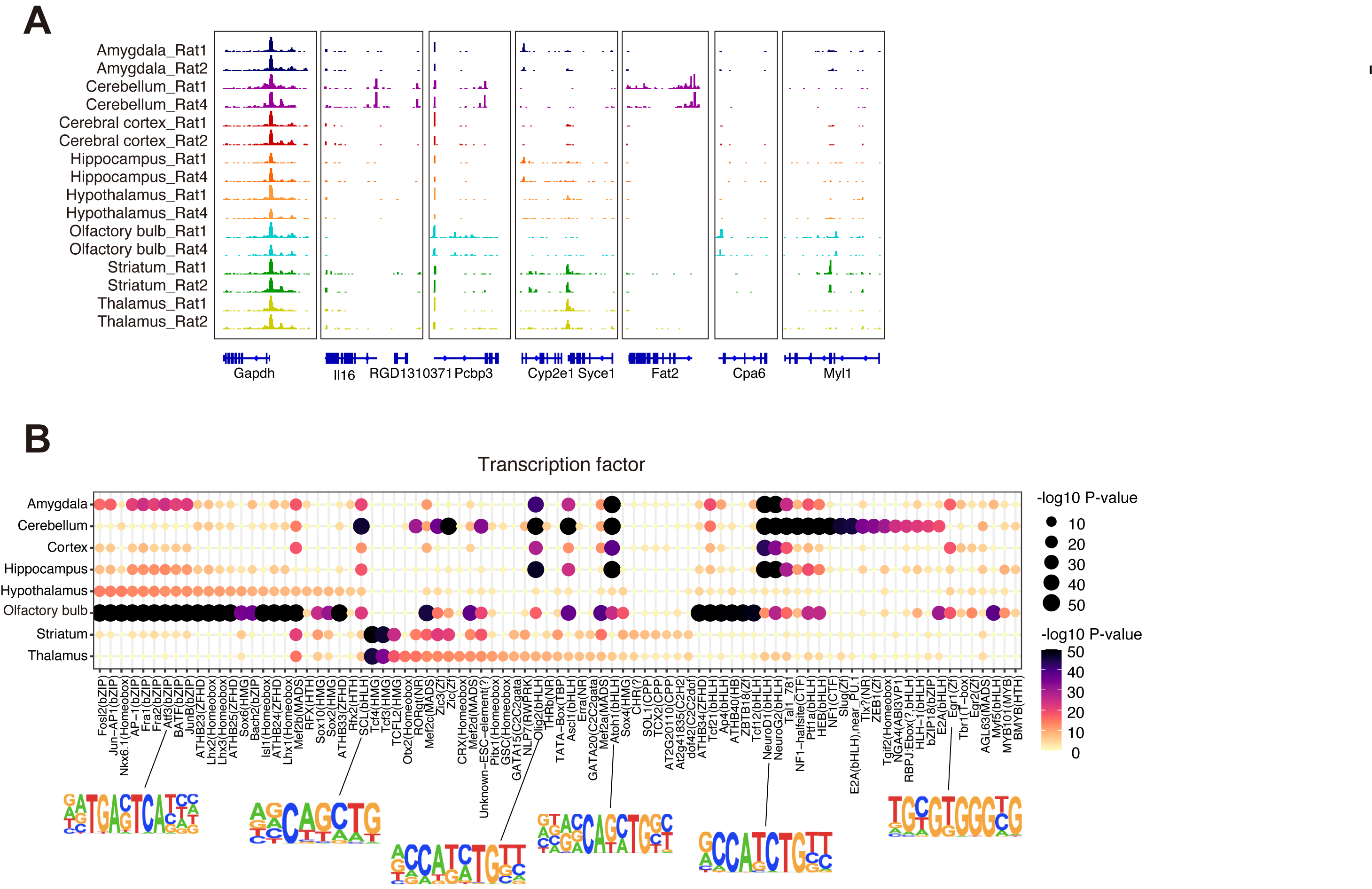

Supplement: Supplementary Figure 1 — The landscape of brain-specific chromatin accessibility and transcription factors. (A) The integrative genomics viewer shows enrichment of ATAC-seq signal for the indicated housekeeping gene (Gapdh) and brain-specific genes. (B) Enrichment of the indicated TF motifs in each tissue. The size and color of each point represent the motif enrichment P-value (–log10 P-value). [file Data_Sheet_1.ZIP › Supplementary/Supplemental Figure 1.tif]
